# Impact of a bronchial genomic classifier on clinical decision making in patients undergoing diagnostic evaluation for lung cancer

**DOI:** 10.1186/s12890-016-0217-1

**Published:** 2016-05-17

**Authors:** J. Scott Ferguson, Ryan Van Wert, Yoonha Choi, Michael J. Rosenbluth, Kate Porta Smith, Jing Huang, Avrum Spira

**Affiliations:** Division of Allergy, Pulmonary, and Critical Care Medicine, Department of Medicine, University of Wisconsin School of Medicine and Public Health, Suite 5233, 1685 Highland Avenue, Madison, WI 53705 USA; Division of Pulmonary and Critical Care Medicine, Stanford University Medical Center, 300 Pasteur Dr, H3143 MC 5236, Stanford, CA 94305 USA; Veracyte, Inc, 6000 Shoreline Ct, #300, South San Francisco, CA 94080 USA; Division of Computational Biomedicine, Department of Medicine, Boston University Medical Center, 715 Albany St, Boston, MA 02118 USA

**Keywords:** Lung cancer, Decision making, Biomarkers, Gene expression, Bronchoscopy, Solitary pulmonary nodule, Clinical utility

## Abstract

**Background:**

Bronchoscopy is frequently used for the evaluation of suspicious pulmonary lesions found on computed tomography, but its sensitivity for detecting lung cancer is limited. Recently, a bronchial genomic classifier was validated to improve the sensitivity of bronchoscopy for lung cancer detection, demonstrating a high sensitivity and negative predictive value among patients at intermediate risk (10–60 %) for lung cancer with an inconclusive bronchoscopy. Our objective for this study was to determine if a negative genomic classifier result that down-classifies a patient from intermediate risk to low risk (<10 %) for lung cancer would reduce the rate that physicians recommend more invasive testing among patients with an inconclusive bronchoscopy.

**Methods:**

We conducted a randomized, prospective, decision impact survey study assessing pulmonologist recommendations in patients undergoing workup for lung cancer who had an inconclusive bronchoscopy. Cases with an intermediate pretest risk for lung cancer were selected from the AEGIS trials and presented in a randomized fashion to pulmonologists either with or without the patient’s bronchial genomic classifier result to determine how the classifier results impacted physician decisions.

**Results:**

Two hundred two physicians provided 1523 case evaluations on 36 patients. Invasive procedure recommendations were reduced from 57 % without the classifier result to 18 % with a negative (low risk) classifier result (*p* < 0.001). Invasive procedure recommendations increased from 50 to 65 % with a positive (intermediate risk) classifier result (*p* < 0.001). When stratifying by ultimate disease diagnosis, there was an overall reduction in invasive procedure recommendations in patients with benign disease when classifier results were reported (54 to 41 %, *p* < 0.001). For patients ultimately diagnosed with malignant disease, there was an overall increase in invasive procedure recommendations when the classifier results were reported (50 to 64 %, *p* = 0.003).

**Conclusions:**

Our findings suggest that a negative (low risk) bronchial genomic classifier result reduces invasive procedure recommendations following an inconclusive bronchoscopy and that the classifier overall reduces invasive procedure recommendations among patients ultimately diagnosed with benign disease. These results support the potential clinical utility of the classifier to improve management of patients undergoing bronchoscopy for suspect lung cancer by reducing additional invasive procedures in the setting of benign disease.

**Electronic supplementary material:**

The online version of this article (doi:10.1186/s12890-016-0217-1) contains supplementary material, which is available to authorized users.

## Background

Lung cancer is the leading cause of cancer death in the United States, with a 5 year survival rate of 17 % and an estimated 158,000 deaths in 2015 [1]. The landmark National Lung Screening Trial (NLST) demonstrated a 20 % relative reduction in mortality through annual low dose computer tomography (CT) in at-risk individuals, though at the expense of 24 % positive screens with a high false positive rate (96 %) [2, 3]. There is a concern that widespread implementation of lung cancer screening programs, along with the increased identification of incidental findings on CT [4], will lead to a substantial increase in the invasive workup of pulmonary nodules and lesions, many of which will be benign after further evaluation.

Bronchoscopy is frequently used to investigate abnormalities discovered on chest CT scans. However, the diagnostic yield of bronchoscopy for lung cancer is between 38 and 83 %, even with the development of navigation and ultrasound technologies [5, 6]. An inconclusive bronchoscopy presents a challenging clinical dilemma of subjecting the patient to the risk of a second invasive diagnostic procedure or the risk of a delayed diagnosis from a watchful waiting approach.

To address this clinical dilemma, a bronchial genomic classifier (Percepta, Veracyte, Inc.) was recently validated in multiple prospective multicenter observational trials to improve the diagnostic sensitivity of bronchoscopy for lung cancer [7, 8]. Based on the airway “field of injury” paradigm in current and former smokers [9], the classifier measures gene-expression profiles in the cytologically-normal bronchial epithelium to distinguish patients with lung cancer versus those with benign diseases of the chest [8]. The bronchial genomic classifier demonstrated a high sensitivity and a negative predictive value (NPV) for lung cancer of 91 % in those with an intermediate (10–60 %) pretest probability of lung cancer and an inconclusive bronchoscopy [7]. A negative bronchial genomic classifier result in this population effectively lowered the probability of lung cancer to less than 10 %, while a positive classifier result maintained the risk category as intermediate.

While the clinical validity and performance of the bronchial genomic classifier have been demonstrated [7, 8], the impact of the classifier on physician decision making has not been directly assessed given that physicians were blinded to the classifier results in those clinical trials. Our overall hypothesis is that a negative genomic classifier result that reclassifies a patient to “low risk” will reduce the rate at which patients will go on for more invasive testing, while not delaying the diagnosis in those patients who have malignant disease.

To begin to test our hypothesis, we selected patient cases from the prospective Airway Epithelial Gene Expression in the Diagnosis of Lung Cancer (AEGIS) trials to construct a case-randomized decision impact survey study. We administered the survey to pulmonary physicians and measured the rate that physicians chose more invasive testing or CT surveillance depending on the results of the bronchial genomic classifier.

## Methods

### Study overview

The Physician Decision Making Impact of the Percepta Bronchial Genomic Classifier (PIONEER) study was a case-randomized, prospective, decision impact study assessing pulmonologist management recommendations in patients undergoing workup for lung cancer who had an inconclusive bronchoscopy. Up to 15 unique patient case vignettes were presented to each experienced pulmonologist; approximately half of the cases contained the classifier result and the rest did not. Which case contained the classifier result and the sequence the cases were presented to each pulmonologist were completely randomized. The pulmonologist’s recommendation for treatment was recorded after each case was reviewed to determine how the classifier results impacted physician decisions.

The primary endpoint was the change in recommendation of invasive procedures with classifier “low risk” (negative) results. The secondary endpoints were the change in recommendations for invasive procedures with classifier “intermediate risk” (positive) results, and the change in rate of invasive procedure recommendations in benign and malignant patients regardless of the classifier results (“low risk” (negative) or “intermediate risk” (positive)).

### Study participants

Eligible study participants were US board-certified pulmonologists who were recruited through Qualtrics’ (Provo, Utah) physician panel. Subjects were enrolled in the study if they indicated they specialized in pulmonology, interventional pulmonology, or pulmonary/critical care medicine, were board certified, practiced pulmonary medicine for at least 2 years post training, performed at least 5 bronchoscopies per month, and indicated that they were likely to use the bronchial genomic classifier after reading a test description. In addition, physicians were asked to correctly identify 2 patient cases as the appropriate lung cancer risk category (low (<10 %), intermediate (10–60 %), or high (>60 %) risk) for lung cancer after reading a brief description including clinical data, smoking history, and nodule characteristics to ensure appropriate physician judgment in assessing cancer risk. The risk levels of these two cases were confirmed using two validated risk calculators [10, 11] and were designed to be on the far ends of the risk spectrum as a quality control metric to ensure physicians completing survey assess risk consistent with ACCP guidance on pulmonary nodule management [12] (see Additional file 1 for case descriptions and risk of malignancy calculations).

### Patient population

Patient cases were selected from the AEGIS trials (AEGIS-1 and −2, NCT01309087 and NCT00746759). These trials were two prospective, multicenter (*n* = 28 centers), observational studies that enrolled 938 current and former smokers without a prior history of cancer who underwent bronchoscopy for suspected lung cancer. The design of these studies have been described in detail [7, 8].

### Patient cases

All cases from the AEGIS data set were extracted in which (i) the pre-bronchoscopy risk of malignancy was either intermediate (10–60 %) as determined by the treating physician, or unassessed, and (ii) the initial bronchoscopy procedure was not diagnostic for cancer. These comprised patients included as part of the training [8] and validation [7] of the classifier. Case details were de-identified and extracted from case report forms and medical charts and described in vignettes. These descriptions included the patient’s clinical presentation and comorbidities, age, gender, smoking history (pack years and years since quitting), other exposure history, CT results (nodule diameter, nodule location, spiculation (when available), presence or absence of adenopathy, longitudinal changes (when available)), PET results (when available), as well as findings from bronchoscopy. Cancer status and bronchial genomic classifier results were not included in the case descriptions.

Two board certified pulmonologists independently reviewed available data for each case. The reviewing pulmonologists were asked to evaluate the case information, including bronchoscopy results, to determine the risk for malignancy and the likelihood of the patient being taken to an additional invasive diagnostic procedure. If only one pulmonologist assessed the case as intermediate risk, the case was sent to a third board certified pulmonologist for adjudication. Cases were included if two pulmonologists made a determination of intermediate risk, and if at least one pulmonologist determined the patient was likely to be taken to an additional invasive procedure.

### Data collection

Physicians were first asked basic information about their clinical practice including type of practice, bronchoscopy techniques used, and how they typically manage patients undergoing workup for lung cancer. Next, physicians were provided with a test description and intended use, clinical validation data and published article [7], and sample test reports (see Additional file 1 for description of test provided to physicians). For each case, physicians were provided with case details detailed above including clinical history, imaging findings, as well as cytology and pathology results from bronchoscopy. For each case that the study physician agreed with the intermediate (10–60 %) risk assessment of the case, the physician was provided or blinded to the bronchial genomic classifier test report in a randomized 1:1 fashion and asked about the next step in their management plan for the patient. Choices were CT surveillance, additional bronchoscopy, trans-thoracic needle biopsy, surgery, or PET scan. If the physician chose PET as the management option, he or she received notice that the PET “yielded indeterminate results (PET scan results did not significantly alter your assessment of malignancy risk for this patient)”. The physician was then prompted to make another management choice.

Participant responses were screened for poor data quality and excluded if: (1) the survey was incomplete, (2) respondents finished the survey in less than 8 min (less than 1/3 of median completion time), or (3) respondents provided identical responses to 5 consecutive questions.

### Description of test reports

Two test results are possible with the bronchial genomic classifier–positive and negative [8]. Because the positive predictive value (PPV) and negative predictive value (NPV) of the classifier are dependent on the pre-test prevalence or probability of malignancy, classifier results are reported in the context of the physician’s pre-bronchoscopy assessed risk of malignancy [7]. For patients with an intermediate pre-test risk of malignancy, a positive result has a PPV of 40 % and is reported as “intermediate risk”. A negative result has an NPV of 91 % and is reported as “low risk” [7].

### Data analysis

The Chi Squared Test was used to assess the primary and secondary endpoints and to compare the rate of invasive procedures in cases reviewed with and without bronchial genomic classifier results. To test whether there were differences in decision making between different physician subgroups, logistic regression models were built assessing the impact of showing low risk (negative) test results across groups. All statistical tests were two-sided at a significance level, 0.05. Statistical analyses were performed using R v3.1.3 software.

## Results

There were 485 physicians screened and 202 physicians that ultimately participated in the study (Additional file 1: Figure S1). Study participants were all US board-certified pulmonologists and represented a broad mix of physicians across geographies and practice type (Table 1). Participants were specialized in at least one of pulmonology (73 %), pulmonary critical care (53 %), or interventional pulmonology (13 %). They had a median (inter-quartile range, IQR) of post-training experience of 14 (12) years and performed a median (IQR) of 10 (12) bronchoscopies monthly for suspected lung cancer.

**Table 1:** Demographics of study participants

Demographic characteristics of study participants (*n* = 202)	
Specialty (*n*, %) (select all apply)	
Pulmonology	147 (73 %)
Pulmonary critical care	108 (53 %)
Interventional pulmonology	27 (13 %)
Years in practice (median, IQR)	14 (12)
Practice affiliation (*n*, %)	
Private group practice	92 (46 %)
University/Academically affiliated practice	44 (22 %)
Practice owned by community hospital or health system	42 (21 %)
Independent practice	20 (10 %)
Government affiliated practice	4 (2 %)
Geography (*n*, %)	
West	60 (30 %)
Northeast	55 (27 %)
South	50 (25 %)
Midwest	37 (18 %)
Bronchoscopies for suspect lung cancer (monthly) (median, IQR)	10 (12)

In total, 36 patients were selected (Additional file 1: Figure S2) and one third of cases were patients with malignant disease (Table 2), which was consistent with the cancer prevalence in the intermediate risk population in the AEGIS studies [7]. There were no significant differences seen between 163 cases reviewed and the 36 cases selected in terms of patient demographics, clinical characteristics, or classifier results (Additional file 1: Table S1). The median (IQR) pack years of these cases was 39 (34) and 19 cases (53 %) had nodules less than 2 cm. PET results were available in 11 of the 36 cases presented. When patient cases were presented to physician participants with genomic classifier results, the actual classifier results were used. The classifier performance, true negative rate and true positive rate within these 36 cases was consistent with that in the AEGIS studies; 50 % of patients with benign disease were down-classified with a negative result and identified as ‘low risk’ and 92 % of patients with malignant disease had a positive result and were identified as ‘intermediate risk’ (Table 3) [7].

**Table 2:** Demographics of patient cases

Variable	(*n* = 36)
Sex	
Male	24 (67 %)
Female	12 (33 %)
Age, median (IQR)	60.5 (23.2)
Race	
Caucasian	32 (89 %)
African American	3 (8 %)
Other	1 (3 %)
Smoking Status	
Former	22 (61 %)
Current	14 (39 %)
Pack-years, median (IQR)	39.0 (33.7)
Lesion size	
< 2 cm	19 (53 %)
2 to 3 cm	5 (14 %)
≥ 3 cm	7 (19 %)
Ill-defined infiltrate	5 (14 %)
Lesion location	
Peripheral	18 (50 %)
Central	7 (19 %)
Both	10 (28 %)
Unknown	1 (3 %)
Lung cancer histology	12 (33 %)
Non-small cell	11 (92 %)
Adenocarcinoma	8 (73 %)
Squamous	2 (18 %)
NSCLC other	1 (9 %)
Small cell	1 (8 %)
Benign diagnoses	24 (67 %)
Infection	8 (33 %)
Resolution or stability	7 (29 %)
Sarcoidosis	1 (4 %)
Other	8 (33 %)

**Table 3:** Patients and case evaluations by cancer status and bronchial genomic classifier results

	Patients	Total case evaluations (*n* = 1523)	
		Not shown classifier results	Shown classifier results
Total	36	787	736
Benign	24 (67 %)	528 (67 %)	491 (67 %)
“Low risk” result	12 (50 %)	252 (48 %)	238 (48 %)
“Intermediate risk” result	12 (50 %)	276 (52 %)	253 (52 %)
Malignant	12 (33 %)	259 (33 %)	245 (33 %)
“Low risk” result	1 (8 %)	16 (6 %)	13 (5 %)
“Intermediate risk” result	11 (92 %)	243 (94 %)	232 (95 %)

In total, 202 physicians provided 1523 case reviews for these 36 patients. Of the 1523 case reviews, 787 (52 %) cases were assessed based on clinical information only without genomic classifier results and 736 (48 %) cases were assessed with genomic classifier results. Table 3 shows the number of patients selected and case evaluations by cancer status and genomic classifier results. Cases were evaluated proportionately to the patient mix, indicating that cases were presented successfully at random without systematic bias.

In order to establish a baseline for physician recommendations, physician responses using clinical information only (blinded to genomic classifier results) were assessed. When blinded to classifier results, physicians recommended invasive procedures for 415 of 787 cases (53 %). This rate did not differ significantly in invasive procedure recommendation for patients ultimately diagnosed with benign or malignant disease (invasive procedure rates of 54 and 50 % respectively, *p* = 0.4). There was also no significant bias in invasive procedure recommendation whether patient cases were associated with intermediate risk (positive) (50 %) or low risk (negative) (57 %) genomic classifier results (*p* = 0.07) since the results were not revealed to physicians. Physician respondents selected PET 16 % of the time; of these ultimately 7 % of these respondents chose CT surveillance, and 9 % chose a second procedure.

**Fig. 1:**
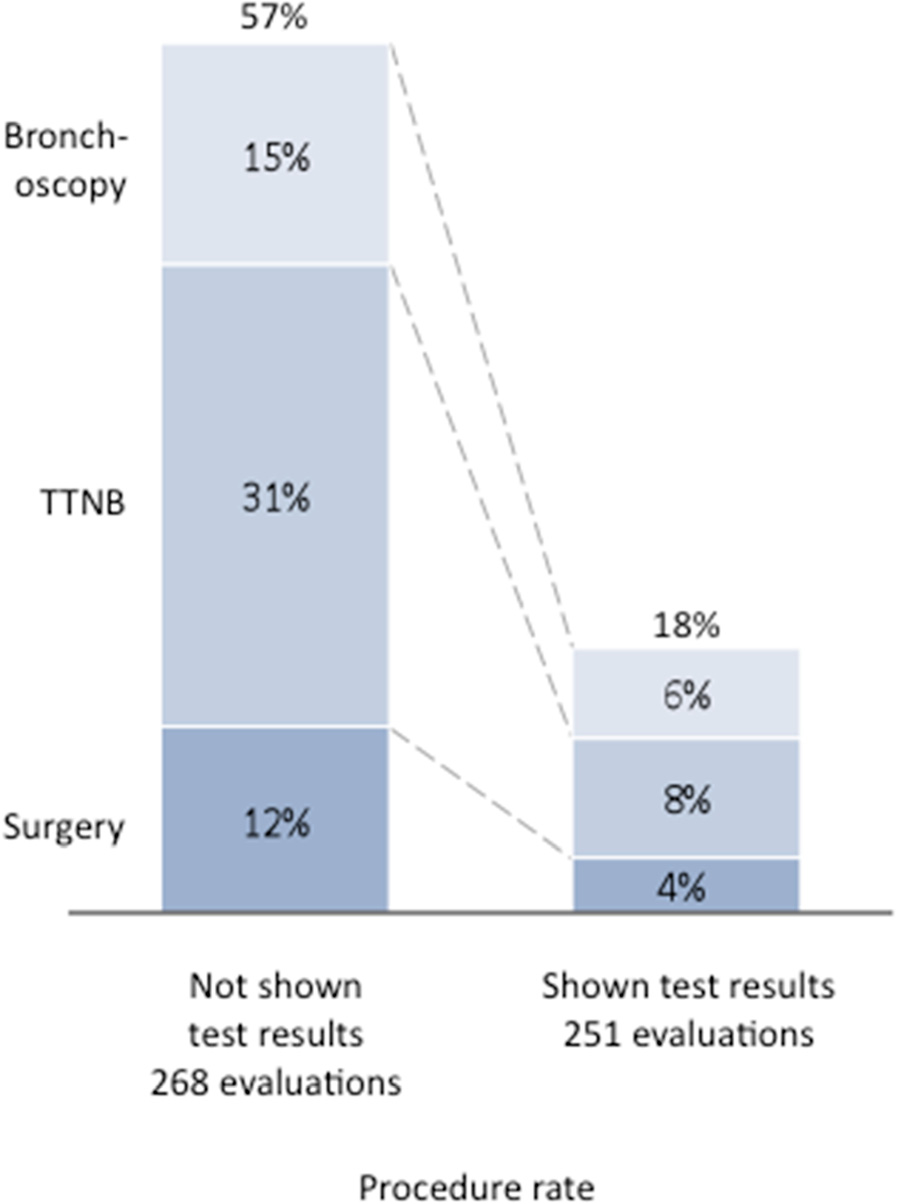
Impact of low risk (negative) bronchial genomic classifier results on decision to take patients to an additional procedure after an inconclusive bronchoscopy. When physicians were presented cases with low risk classifier results (*n* = 251), 18 % of the time they recommended a procedure, a three-fold reduction from when physicians were presented blinded cases associated with low risk classifier results (57 % invasive procedure rate, *n* = 268, *p* < 0.001)

To examine the primary study endpoint of the impact of low risk (negative) genomic classifier results on reducing invasive procedure recommendations, the rate of procedure recommendations was compared when physicians had access to clinical information alone versus access to genomic classifier results as well. Invasive procedure recommendations were reduced from 57 % (*n* = 154 cases out of 268 cases) when physicians only had access to clinical information to 18 % (*n* = 44 cases out of 251 cases) when the low risk (negative) results were also reported (Fig. 1), a three-fold decrease (*p* < 0.001). Figure 1 further details the rate of the procedure types that physicians recommended. No significant differences in decision making were observed between pulmonologist type (interventional vs other, *p* = 0.46) or those who performed higher (>10) versus lower (≤10) volume of bronchoscopies for lung cancer per month (*p* = 0.89) (Additional file 1: Tables S2 and S3).

**Fig. 2:**
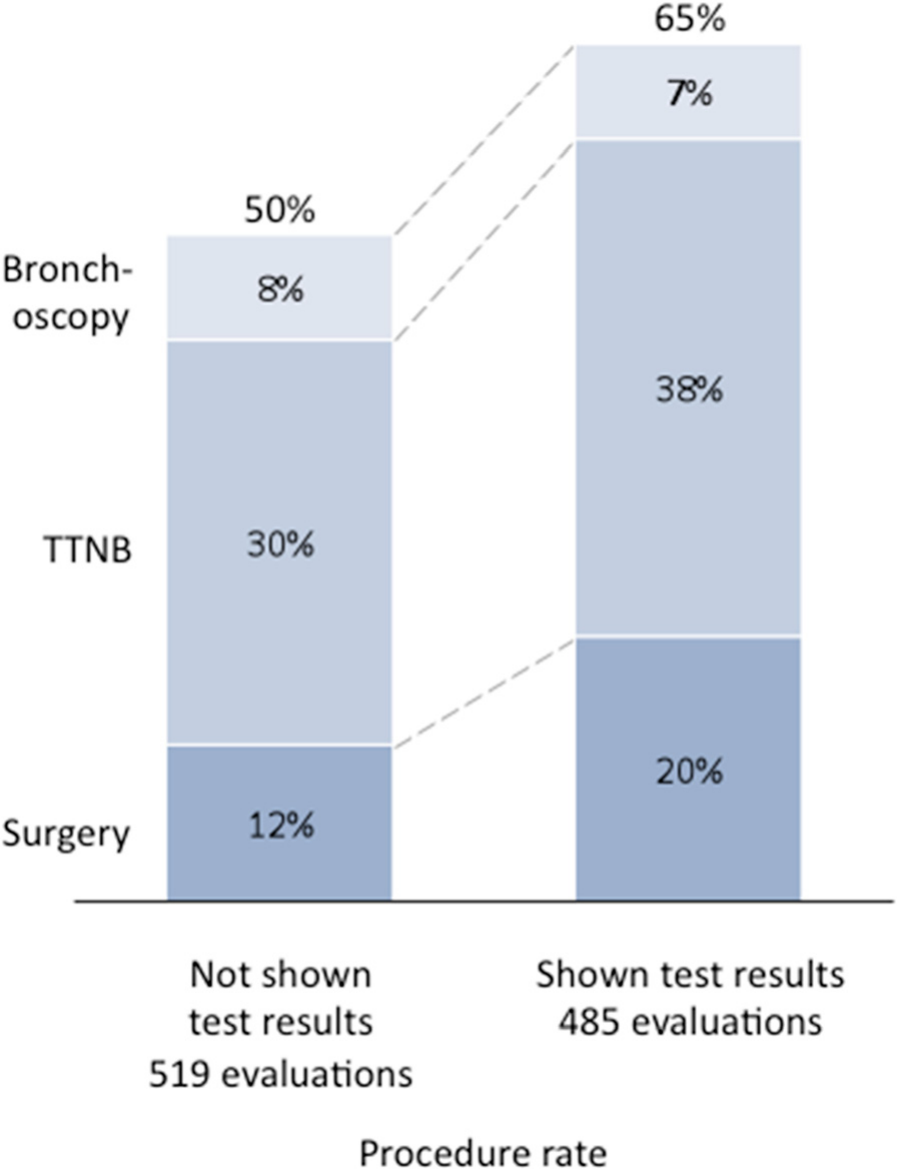
Impact of intermediate risk (positive) bronchial genomic classifier results on decision to take patients to an additional procedure after an inconclusive bronchoscopy. When physicians were presented cases with intermediate risk classifier results (*n* = 485), 65 % of the time they recommended a procedure, a 15 percentage point increase from when physicians were presented blinded cases associated with intermediate risk classifier results (50 % invasive procedure rate, *n* = 519, *p* < 0.001)

As a secondary endpoint, we investigated the impact of intermediate risk (positive) classifier results on physician recommendations and similarly compared when physicians had access to clinical information alone versus genomic classifier results as well. Invasive procedure recommendations were increased from 50 % (*n* = 261 cases out of 519 cases) when physicians only had access to clinical information to 65 % (*n* = 314 cases out of 485 cases) when the intermediate risk (positive) results were also reported (Fig. 2) (*p* < 0.001). Figure 2 further details the rate of the procedure types that physicians recommended.

**Fig. 3:**
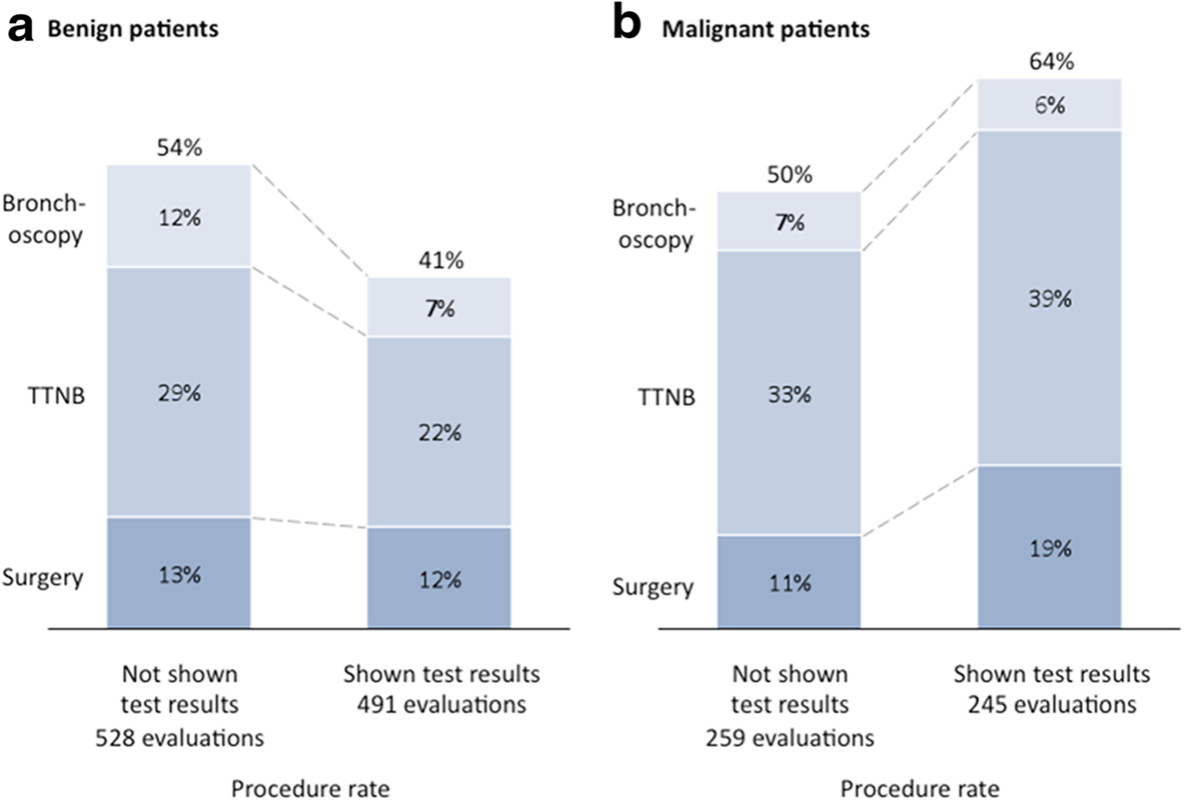
Rate of procedures by physicians for all benign or malignant patients when not shown vs. shown bronchial genomic classifier results. **a** There was an overall reduction in physician decision to take benign patients to procedures when presented with classifier results (54 to 41 %, *p* < 0.001). **b** In malignant patients, there was an overall increase in physician decision to take to a procedure when presented with classifier results (50 to 64 %, *p* = 0.003)

As an additional secondary endpoint, we investigated the overall impact of the genomic classifier results (both low risk (negative) and intermediate risk (positive) results) on physician decision making in patients ultimately diagnosed with either malignant or benign disease (Fig. 3). In the benign group, there was an overall reduction in invasive procedure recommendations observed when classifier results were reported. The rate of invasive procedure recommendation significantly decreased from 54 % (285 cases out of 528 cases) to 41 % (201 cases out of 491 cases) (*p* < 0.001). For the malignant group, there was an overall increase in invasive procedure recommendations when the classifier results were reported. The rate of invasive procedure recommendation increased significantly from 50 % (130 cases out of 259 cases) to 64 % (157 cases out of 245 cases) (*p* = 0.003). Management recommendations within benign and malignant groups segmented by classifier result type (low vs intermediate) are shown in Additional file 1: Figure S3.

## Discussion

In this study, we sought to determine the potential clinical impact of a bronchial genomic classifier on decision making in patients at intermediate pretest risk of lung cancer whose bronchoscopy was inconclusive. We used a clinical decision impact survey of 202 physicians evaluating 36 cases (1523 total case evaluations) randomized to the availability or absence of the bronchial genomic classifier results. Without classifier results, we found that physicians recommended an additional invasive procedure 53 % of the time. This is consistent with other observational studies that have examined management of indeterminate pulmonary lesions and suggest that decision making by physicians in this survey is reflective of the real world. In a multi-center observational study of procedure use of community pulmonologists on patients with indeterminate pulmonary nodules, physicians performed biopsy in 54 % of patients (202 of 307) [13]. In the AEGIS data set, in patients at intermediate pretest risk of malignancy with an inconclusive bronchoscopy, physicians performed a biopsy in 53 % of patients (62 of 117 patients) [14]. In a study assessing guideline concordance in evaluation of pulmonary nodules, patients with nodules larger than 8 mm underwent non-surgical biopsy 33 % of the time and surgical resection 12 % of the time [15].

We found that the availability of bronchial genomic classifier results significantly altered physician decision making. When low risk (negative) classifier results were shown to physicians, there was a 3-fold reduction in the rate of invasive procedure recommendations as compared to cases when the classifier results were not shown. These results suggest that the use of the genomic classifier in the intermediate pretest risk population may reduce the rate of invasive testing among patients with benign lung disease.

Additionally, we found that there was an increase in procedure recommendations from 50 to 65 % when intermediate risk results were shown. This was a surprising finding, as intermediate risk results confirmed the physicians’ initial pre-test risk assessment and thus procedure recommendation rate would have been expected to be similar. We speculate that physicians, when presented with an additional data point that does not reduce probability of malignancy, may tend towards a more aggressive approach rather than surveillance. These findings highlight the importance of physician education in interpreting results of the classifier in terms of its negative and positive predictive values.

We also found that there was an overall significant reduction in invasive procedure recommendations for patients with an ultimate benign diagnosis when the bronchial genomic classifier results were shown. Conversely, in patients with an ultimate diagnosis of cancer, there was an increase in invasive procedure recommendations overall when the results of classifier were shown. These results are ultimately due to the prevalence of disease in low risk (negative) vs. intermediate risk (positive) classifier results. Those individuals with cancer almost always had intermediate risk (positive) results, while those without cancer were split between low-risk (negative) and intermediate risk (positive) results.

Our study does have some limitations that should be addressed. First, physicians were provided a summary of the patient’s clinical presentation (age, gender, comorbidities), smoking history (pack years, years since quitting) and exposure history, physical exam findings, lesion details from the CT and PET reports (nodule diameter, location, presence of adenopathy), but did not have direct access to the CT image, which can impact decision making in this setting. However, based on the information provided, physicians would have had enough information to calculate risk from several pulmonary nodule risk calculators, including the Veteran Affairs [16] and Mayo [17] models. An additional related caveat is that physicians who chose PET as the management option were notified that the PET results were indeterminate and did not significantly alter the risk of malignancy, prompting them to make another management choice. Inclusion of this option was intended to simulate real-world clinical decision making for the participant. PET scanning is often done pre-bronchoscopy as part of the pre-test risk assessment in settings where physicians feel it will add value. In the AEGIS trial, PET was performed and data available in 11 of 36 cases included in this study. However, we recognize that some physicians may elect to choose PET following an inconclusive bronchoscopy, a choice that was made 16 % of the time within the survey.

Second, this was a clinical decision impact study presented in survey form and not a clinical trial or registry. As such, it can only approximate clinical utility using responses from a population who may have some form of selection bias for entering into this type of study. We also recognize that decisions made in a survey may not accurately reflect those made at point of care. However, decision making in proceeding to invasive procedures when blinded to classifier results in our study was similar to real world observational studies [13-15]. Further, decision impact studies have successfully evaluated changes in clinical decision-making and healthcare utilization associated with novel cardiovascular [18, 19] and oncology [20–24] diagnostic tests. The concordance between clinician treatment plans and actual clinical management has been shown to be between 80 and 85 % [19, 20], suggesting that this methodology is a valid tool to predict the ultimate utility of a diagnostic test.

Third, we recognize that clinical decision making is ultimately modulated by patient preferences which cannot be captured by this survey. However, the rate of invasive testing in our survey by physicians blinded to the classifier results was comparable to that seen in the observational multicenter AEGIS trial suggesting that clinical decision making in our survey mirrors that seen in clinical practice [14].

There are a number of key strengths to our study design. First, the clinical impact survey leveraged actual cases from the multicenter prospective AEGIS trials [7, 8], likely reflecting the general mix of patients that might benefit from the classifier. In particular, we limited the survey to those patients whose bronchoscopy was inconclusive and where the pretest risk of cancer was intermediate, the clinical setting in which physicians have the greatest uncertainty about subsequent invasive testing [25]. Our case selection process included review and adjudication of the available case data by board certified pulmonologists to ensure that the case represented an intermediate risk of malignancy based on the available data. Additionally, we reported the actual results of the classifier for the patients from that trial in a randomized fashion, enabling us to model the impact of the classifier on clinical decision making. Third, we intentionally avoided the more conventional matched-pair design where each physician will be presented with a case first without the classifier information and then with the classifier information and asked for their treatment recommendation for each scenario consecutively. Instead, for each physician being surveyed, which case contains the classifier information as well as the order the cases were presented were completely randomized. This potentially reduced cognitive bias due to the anchoring effect: the tendency to keep one’s belief or revise one’s belief insufficiently when presented with new evidence (in this case the genomic classifier result) [26, 27].

## Conclusions

The results from this clinical decision impact study suggest that a bronchial genomic classifier has the capacity to change decision making in patients suspected of lung cancer with an inconclusive bronchoscopy. We found that practicing physicians who are provided low risk results from the classifier recommended significantly fewer invasive procedures among intermediate pretest risk patients. Given the growing unmet medical need to improve the evaluation and management of lung lesions found on chest imaging, the bronchial genomic classifier has the potential to reduce the burden of additional invasive diagnostic procedures in patients with benign disease.

### Ethics approval and consent to participate

The study was reviewed and approved by an independent institutional review board (Western Institutional Review Board, Puyallup, WA, Study Number 1157098). The study was administered via an online questionnaire administered by Qualtrics. The study was conducted in compliance with good clinical practice (GCP) with the applicable regulatory requirements. The survey process was conducted by personnel trained in the protection and confidentiality of protected health information (PHI). All study physicians were provided electronic written consent for study participation. Physicians were de-identified by the survey administrator before data was provided to the authors.

### Consent for publication

Not applicable.

### Availability of data and materials

Raw survey data is available at http://www.veracyte.com/pioneerdata.

## Additional file

Additional file 1: Supplementary materials. (DOCX 194 kb)

## Abbreviations

AEGIS: airway epithelial gene expression in the diagnosis of lung cancer
CT: computed tomography
EBUS-TBNA: endobronchial ultrasound transbronchial needle aspiration
GCP: good clinical practice
IQR: interquartile range
NLST: National Lung Screening Trial
NPV: negative predictive value
PET: positron emission tomography
PHI: patient health information
PIONEER: physician decision making impact of the percepta bronchial genomic classifier
PPV: positive predictive value
