# Supplementary material for: Impact of a bronchial genomic classifier on clinical decision making in patients undergoing diagnostic evaluation for lung cancer
Source: BMC Pulm Med. 2016 May 17;16:66. doi: 10.1186/s12890-016-0217-1 (PMC4869188; doi:10.1186/s12890-016-0217-1)
Supplement: Supplementary file 1 — Supplementary materials. (DOCX 194 kb) [file 12890_2016_217_MOESM1_ESM.docx]

Additional file

Impact of a bronchial genomic classifier on clinical decision making in patients undergoing diagnostic evaluation for lung cancer

J. Scott Ferguson*, Ryan J. Van Wert†, Yoonha Choi‡, Michael J. Rosenbluth‡, Jing Huang‡, and Avrum Spira§

**Figure S1**


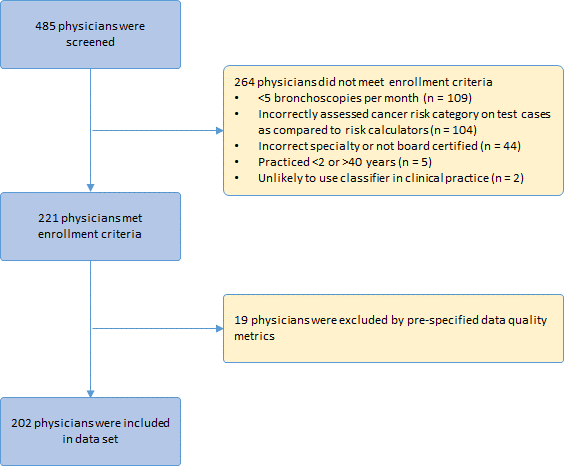


**Figure S1:** Selection of the 202 physician participants in the study. A total of 485 physicians entered the study via an e-mail invitation. Of these, 221 physicians met the enrollment criteria. Nineteen physicians were excluded by pre-specified data quality metrics (either a survey completion time of less than 8 minutes or physician did not vary response for 5 or greater questions in a row).

**Figure S2**


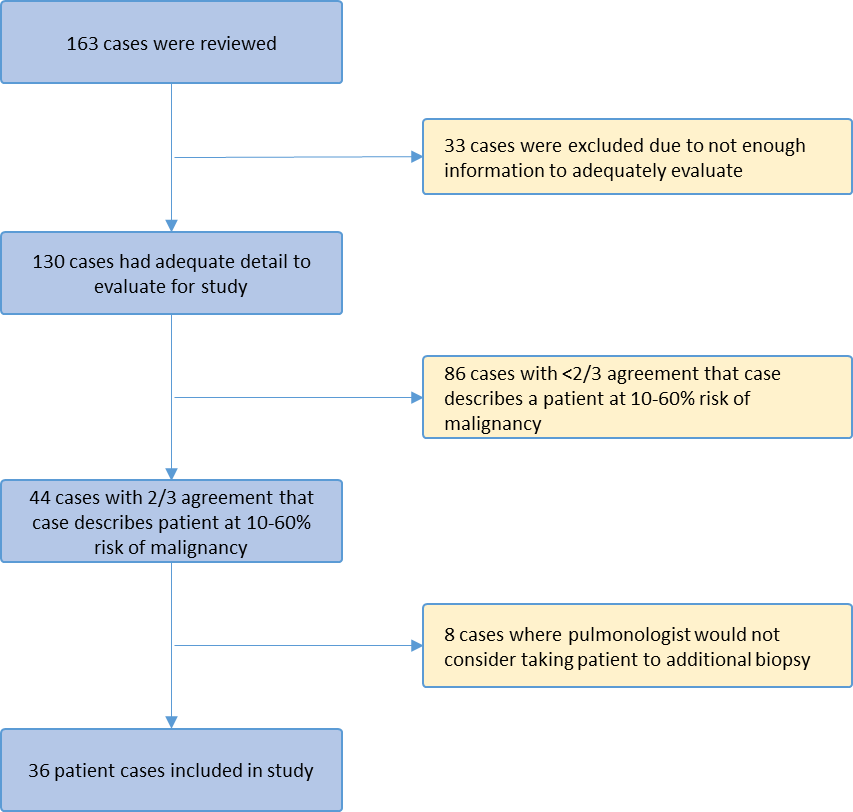


**Figure S2:** Selection of the 36 cases included in the study. A total of 163 cases were reviewed. These are comprised of 103 patients from the AEGIS-1 and -2 validation (Silvestri, et al 2015) with inconclusive bronchoscopies and either intermediate pre-test risk of malignancy or risk unassessed, as well as 60 patients from AEGIS-1 that were used for training (Whitney, et al 2015) in these same pre-test risk classification categories. 33 cases were excluded due to not enough information to adequately evaluate. 86 cases had discordance in pre-test risk assessment given the case description and were excluded. 8 cases when at least one of the reviewing pulmonologists would not consider taking the patient to an additional biopsy were also excluded. No significant differences were seen in the patient demographics between the 163 cases reviewed and the 36 cases selected (Table S1).


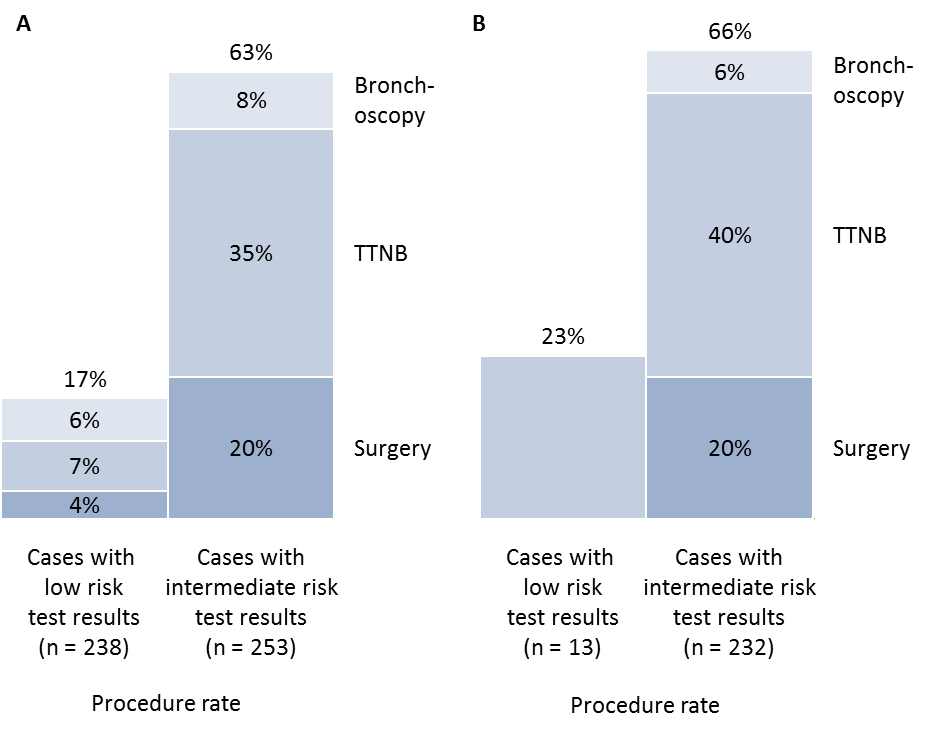
**Figure S3**

**Figure S3. Impact of genomic classifier test results on decision to take patients to an additional procedure after an inconclusive bronchoscopy.**
**A. Decision making in benign patients.** The 238 cases with low risk test results shown were recommended to go to a procedure 17% of the time by physicians. The 253 cases with intermediate risk test results shown were recommended to go to an additional procedure 63% of the time by physicians. **B. Decision making in malignant patients.** The 13 cases with low risk test results shown were recommended to go to a procedure 23% of the time by physicians. The 232 cases with intermediate risk test results shown were recommended to go to an additional procedure 66% of the time by physicians.

**Table S1. Patient demographics, clinical characteristics, and classifier results of the patient cases reviewed and selected for the study.**

**
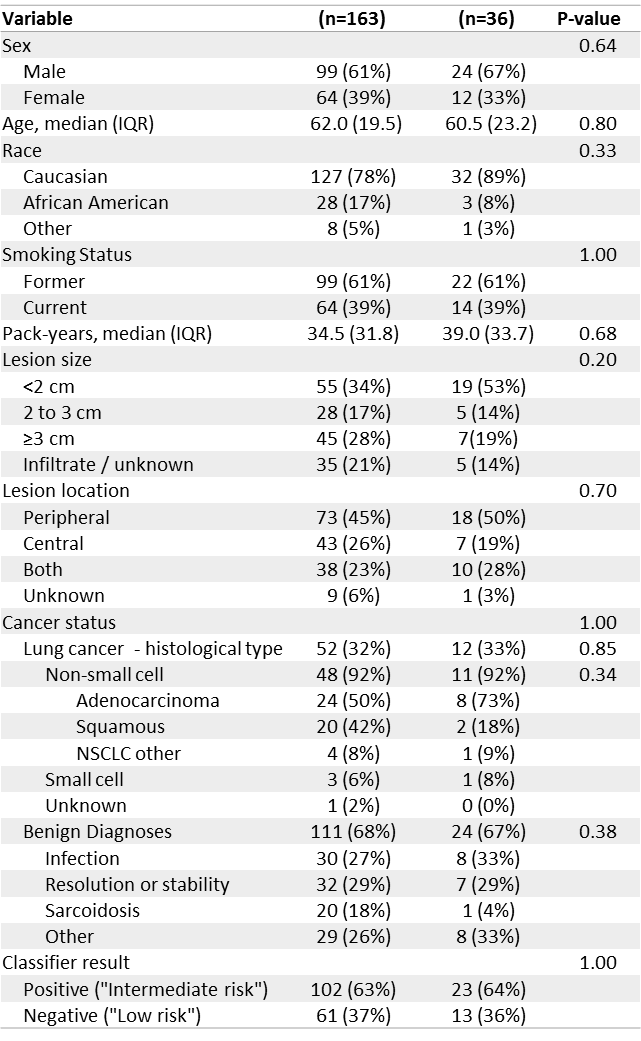
**

**Table S2. Impact of classifier on procedure recommendations for cases with low risk (negative) test results, analyzed based on whether pulmonologist was interventionalist**


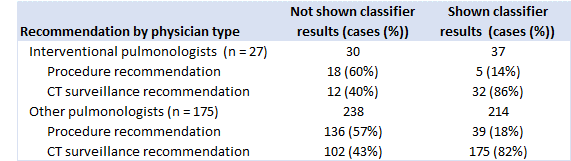


**Table S3. Impact of classifier on procedure recommendations for cases with low risk (negative) test results, analyzed based on physician bronchoscopy volume**


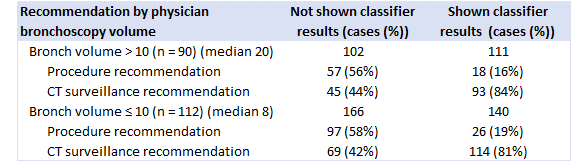


**Vignettes used in survey screener**

Physicians were asked two clinical vignettes in the screener as a quality control metric. Physicians needed to correctly select the appropriate risk category (low, <10%; intermediate (10-60%; high, >60%) in order to enroll in the study and complete the survey. Two models were used to determine risk based on the clinical factors described in the cases. Vignette 1 was assessed as high risk (Gould / VA model: 91% and McWilliams / Brock University Model: 83%). Vignette 2 was assessed as low risk (Gould / VA model: 1% and McWilliams / Brock University Model: <1%).

Vignette 1: Consider the following patient: An 82 year old male presents with a pulmonary mass.  The patient has 50 pack years of smoking history and quit smoking 2 years ago.  He currently has emphysema.  The lesion is 40 mm, solid, spiculated, and in the right upper lobe.   What would you estimate this patient's risk of malignancy to be?

Vignette 2: Consider the following patient: A 55 year old female presents with a solitary pulmonary nodule.  The patient has 5 pack years of smoking history and quit smoking 15 years ago.  She has no other identified lung diseases.  The nodule is 4 mm, solid, not spiculated, and in the right lower lobe. What would you estimate this patient's risk of malignancy to be?

**Representative cases used in survey**

Two representative cases provided to physicians in the survey are provided below, along with the ultimate diagnosis of the patients. Ultimate diagnosis was not presented to the physicians in the survey.

**Case 1 (ultimate benign diagnosis)**

**Clinical presentation:**

- 50 year old white female
- Current smoker with a 23 pack year history
- Presents with cough, suspicious X-ray

**CT Scan:**

- CT shows a nodule 1.7cm x 1.7cm in size in RUL that is concerning for neoplasm
- Also identified are multiple smaller lesions that appear inflammatory in medial RML, lingula, LUL
- No lymphadenopathy

**Bronchoscopy results:**

- Normal airways
- RUL lesion could not be localized via EBUS
- Right BAL: acute inflammation, no evidence of malignancy or organisms

**Case 2 (ultimate malignant diagnosis)**

**Clinical presentation:**

- 54 year old white female
- Current smoker with a 40 pack year history who began smoking at age 14
- No personal history of cancer
- Presents with shortness of breath and cough

**CT Scan:**

- 1 x 1.3 cm left lower lobe peripheral lesion with lobulated but smooth margins
- Predominant centrilobular emphysema

**Bronchoscopy results:**

- TBNA- no evidence of malignancy
- Brushing- specimen insufficient for interpretation
- TBBX- mildly atypical cells, favor reactive change
- BAL- no evidence of malignancy

**Bronchial Genomic Classifier description**

Physicians were provided with a description of the test, its intended purpose, a summary of the clinical data, and a link to the pivotal clinical validation paper (Silvestri, et al, 2015). The description of the classifier provided is as follows:

**Description:** The Percepta Bronchial Genomic Classifier identifies current or former smokers who are at low risk for lung cancer following an inconclusive bronchoscopy. These low risk patients may be considered for CT surveillance and avoid unnecessary invasive procedures.


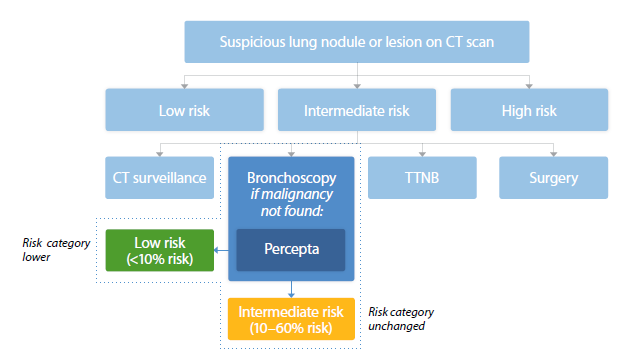
In current or former smokers with lung cancer, gene expression alterations occur throughout the airway. Percepta analyzes these genomic alterations using epithelial cells collected from two standard brushings of the mainstem bronchus during bronchoscopy. It does not require a sample from the nodule or lesion.

If the patient is deemed to be intermediate risk by the physician prior to the bronchoscopy and use of the Percepta test, Percepta will show one of 2 results in this patient population:

- Low Risk (<10% risk): the patient has been reclassified from an Intermediate to Low Risk category. Thus, the patient is at low risk for lung cancer and monitoring with CT surveillance can be considered.

- Intermediate Risk (10-60%): the patient's risk category is unchanged. Thus, the physician should proceed with next steps in the diagnostic process as previously planned.

*Link to test reports provided here*

**Clinical validity:** The test has been clinically proven in three multicenter studies with over 1,000 patients enrolled across more than 30 medical centers. As demonstrated in the recent New England Journal of Medicine publication of its clinical validation studies, Percepta has a high sensitivity and high negative predictive value.

**Test specifications and performance:**

| **Intended test population** | Patients with an identified pulmonary nodule or lesion |
| --- | --- |
|  | History of smoking, no previous history of other cancers |
|  | Bronchoscopy did not identify malignancy (test only run if bronchoscopy inconclusive) |
| **Sample Collection** | 2 standard brushings from the mainstem bronchus taken during the initial bronchoscopy |
|  | Does not require a sample from the nodule or lesion. |
| **Genomic Test performance for patients with a non-diagnostic bronchoscopy (29% cancer prevalence in study population):** |  |
| Negative Predictive Value (NPV), low risk result (in an intermediate risk patient) | 91% |
| Positive Predictive Value (PPV), intermediate risk result (in an intermediate risk patient) | 40% |
| Sensitivity / specificity of bronchoscopy plus genomic test | 97% / 47% |
| Sensitivity / specificity of genomic test alone | 89% / 47% |
| **Subpopulation performance** | Sensitivity and specificity independent of nodule and lesion size and location (peripheral vs central), lung cancer stage, lung cancer type |
| **Data supporting performance** | Three independent, multi-center clinical studies, with over 1,000 patients combined, published in two peer-reviewed journals, including the New England Journal of Medicine |

*Link to Silvestri et al NEJM 2015 provided here*
